# Patient perceptions in quality of care: report from university veterans clinic

**DOI:** 10.1186/s12903-019-0971-6

**Published:** 2019-12-03

**Authors:** Nayanjot K. Rai, Heidi Tyrrell, Clifton Carey, Tamanna Tiwari

**Affiliations:** 1 Department of Community Dentistry and Population Health, University of Colorado School of Dental Medicine, Mail Stop F843, 13065 East 17th Avenue, Room 104D, Aurora, CO 80045 USA; 2 Heroes Clinic and Veterans Outreach Coordinator, University of Colorado School of Dental Medicine, Mailstop F839, 13065 East 17th Avenue Room 104H, Aurora, CO 80045 USA; 3 Department of Craniofacial Biology, Translational Research, University of Colorado School of Dental Medicine, Mail Stop 8310, 12800 East 19th Avenue, Bldg RC1-North, Room 2106, Aurora, CO 80045 USA; 4 Department of Community Dentistry and Population, Center for Oral Disease Prevention and Population Health Research, University of Colorado School of Dental Medicine, Mail Stop F843, 13065 East 17th Avenue, Room 104F, Aurora, CO 80045 USA

**Keywords:** Provider empathy, Perceptions of care, Quality of care, Patient satisfaction

## Abstract

**Background:**

The Heroes Clinic is a unique dental clinic housed at the University of Colorado School of Dental Medicine that offers military veterans dental care at no or minimal cost. The aim of this study is to collect patient feedback on their perception of the quality of care they receive at the Heroes clinic.

**Methods:**

A cross-sectional study design was used to gather patient feedback on empathy and quality of care using Service Quality Measures (SERVQUAL) and Dental Satisfaction Questionnaire (DSQ) frameworks. Mean scores were calculated to determine the average of positive or negative responses. Fisher’s exact test was conducted to test any significant differences between the patients’ perception of quality of care they receive at the Heroes clinic (outcome variable) and the SERVQUAL and DSQ independent variables.

**Results:**

One hundred and seventy-seven veterans responded to the survey with a response rate of 35%. Over 50% of patients were between the ages of 20–35 years and 63% were students. The mean scores demonstrated high levels of all variables. Bivariate analysis for SERVQUAL data determined that veterans agreed to conditions demonstrated by four scales of empathy and all scales of responsiveness (*p* < 0.05). DSQ bivariate analysis revealed that veterans agreed to conditions demonstrated by four scales of quality of care, two scales of pain management, one scale of accessibility, and also general satisfaction pertaining to the received dental care (*p* < 0.05).

**Conclusions:**

Heroes clinic has provided quality dental care to veterans as attested by the patients.

## Background

Evaluation of healthcare quality has emerged as a significant matter in the care process and it has been recognized that patient feedback is an essential component of these evaluations [1, 2]. The healthcare industry is shifting towards a consumer-oriented approach where the dentists are considered as service providers and the patients as customers [3]. The patient-clinician relationship is considered therapeutic in nature as the clinical, interpersonal skills necessary to ensure patient safety, care and comfort included in treatment planning generate not only patient satisfaction but also gratitude [4]. It is recognized that patients cannot assess the medical competence of the dentist or clinician, but their experience towards the process of care underlines their perception regarding the quality of care and its improvement [5]. Patient satisfaction is a widely accepted measure of healthcare efficiency, is crucial in providing information on patient expectations, and influences the dental schools’ pattern for service utilization [5, 6]. It has been seen that highly satisfied patients present with better compliance and less anxiety and pain perception [5, 7]. This study will provide an insight into the military veterans’ experience, satisfaction and, perceptions of quality of dental care they receive at the Heroes clinic housed at the Colorado University (CU) School of Dental Medicine (SDM).

The veteran population report high comorbidities such as cardiovascular disease and chronic pain [8]. It has been seen that deployment to war can profoundly affect a veteran’s health and personal life, carrying the risk of long-term physical, psychological, and social impairments [9]. Dental injuries and dental diseases are listed as some of the topmost concerns in veterans due to a combat environment and difficult living conditions [9]. In addition, dental utilization among veterans is low due to several reasons, such as lack of insurance and higher comorbidities or dental anxiety [10]. The Veterans’ Administration (VA) provides dental care to veterans classified as 100% disabled and those with a service-connected oral injury.

### Background on heroes clinic

Established in 2014 at the CU-SDM, the Heroes clinic provides free dental care to student veterans CU’s Denver and Anschutz Medical campuses to help them to transition into the workforce with a healthy smile. Later, the Heroes clinic expanded to provide dental care to veterans at all four CU campuses and four local universities in the state. The Heroes clinic aims to provide all the dental care to the military veterans based on their needs and values while developing a trusting relationship with student providers and clinic faculty. Furthermore, the Heroes clinic provides dental care to homeless veterans striving for employment and veterans who do not qualify for Medicaid or do not have any form of insurance and struggled to afford dental care or find providers.

A workshop was held in 2014 for all faculty to orient them towards the special needs of the veteran population. It was presented by the University’s disability director and four veteran patients with Hidden Disabilities, which includes various forms of emotional disabilities, Traumatic Brain Injury, and Post Traumatic Stress Disorder. The workshop underscored that the veterans’ past must be considered and new experiences should be created that allows them to receive dental care while feeling safe and non-judged. For example, the use of a weighted or a “thunder” blanket has proved useful for some veterans, who had anxiety related to dental treatment. Thunder blanket is a heavy blanket or a lead apron, which is placed on the top of the patient lying on the chair that helps to reduce anxiety and feeling of vulnerability in many patients. Other adaptions used in the Heroes clinic are location of the patients in the clinic; matching gender of the provider with the gender of the patient to accommodate those patients with a history of sexual abuse; and scheduling a patient “protected” by a wall or the area where the patient can observe other people entering the area. Furthermore, the scope of treatment provided in the Heroes Clinic is not limited to restoring teeth. An extensive network of veteran providers across Colorado in all disciplines of healthcare has been created. In addition, referral networks have been built for housing resources, veteran benefits, Volunteers of America, medical providers, service animals, and more recently Traumatic Brain Injury and Post Traumatic Stress Disorder specialty clinics. These resources are offered to all veterans, which may help to improve their day-to-day lives.

In addition to the workshop, senior dental student providers undergo an orientation before they start the rotation. This includes instructions on how to communicate with the patients, the importance of timeliness of treatment, the importance of detailed explanation of treatment modalities and emphasis on special needs of the veteran patients, including managing anxiety and treatment-related apprehensions.

The objective of this study was to collect patient feedback on their perception of the quality of care they receive at the Heroes clinic.

## Methods

This study was approved by the Colorado Multiple Institutional Review Board (COMIRB: 17–0968). All participants gave signed consent for participating in the study. The consent form was sent via email along with the survey link to the veterans. A cross-sectional survey was used to gather the data using Service Quality Measures [11] (SERVQUAL) and Dental Satisfaction Questionnaire (DSQ) frameworks [12].

SERVQUAL is used to measure perceptions of care service quality. Although developed to be used in the field of marketing, it was later modified to be used in medicine [13] and dentistry [14]. Originally, SERVQUAL has four measures: responsiveness, empathy, reliability and tangibles. Carman (1990) confirmed that the instrument may be adapted for use in any industry within the stated guidelines [15]. SERVQUAL was therefore modified to the attendance at the CU Heroes clinic. It included two measures, Empathy and Responsiveness. Empathy measures three attributes including, provider’s communication skills, attention to detail, and knowledge about the needs of their patients. Responsiveness measures the provider’s ability to help the patients, response level to the patients’ requests, and skill of performing standard procedures (Table 1). The DSQ framework includes four measures, Access to care, Availability of necessary providers and services/Convenience of location and hours of the clinic to the patients, Pain management, and Quality of care provided at the clinic (Table 1). Both the SERVQUAL and DSQ frameworks measured the level of attributes using a 5-point Likert scale.

**Table 1:** Description of SERVQUAL and DSQ

SERVQUAL	Items**
Empathy (5 sub-scales)	1. My dental student clearly explained all treatment 2. The assistant director gave explanation related to administration procedures. 3. The dental student gave personal attention in listening to the patient’s complaint. 4. Assistant director gave personal attention to registration procedures. 5. The dental student was knowledgeable regarding the patient’s needs for the treatment.
Responsiveness	1. The assistant director responded promptly to long time waiting. 2. The assistant director responded promptly to issues or concerns related to care. 3. The assistant director responded promptly to issues or concerns related to delays in care.
DSQ	Items
General Satisfaction	There are things about the dental care I receive at the CU Heroes Clinic that could be better.*
Quality of care	1. Colorado University Heroes Clinic dental students are very careful to check everything when examining their patients. 2. Colorado University Heroes Clinic dental students always treat their patients with respect. 3. Colorado University Heroes Clinic dental students are not as thorough as they should be. * 4. Dental students usually explain what they are going to do before they begin treatment. 5. The Colorado University Heroes Clinic is very modern and up to date. 6. Dental students should do more to keep people from having problems with their teeth.*
Pain Management	1. Sometimes I avoid going to the dental student because it is so painful. * 2. Colorado University Heroes Clinic dental students should do more to reduce pain. * 3. I am not concerned about feeling pain when I go for dental care at Colorado University Heroes Clinic.
Availability/Convenience	1. One of the reasons I come to the Colorado University Heroes Clinic is because there are not enough dentists in my area.* 2. Colorado University Heroes Clinic Dental Clinic is very conveniently located.
Accessibility	1. It is hard to get an appointment at the Colorado University Heroes Clinic for dental care right away.* 2. Office hours at the Colorado University Heroes Clinic are good for most people.

*SERVQUAL* Service Quality Measures

*DSQ* Dental Satisfaction Questionnaire

* Denotes negative direction of scoring

**Five response choices accompanies each item: strongly agree, agree, not sure, disagree and strongly disagree. These responses are coded 1,2,3,4,5 for items with negative direction of scoring and 5,4,3,2,1 for those with positive scoring. A higher score on all scales indicates greater satisfaction

The survey was conducted using an electronic platform, Redcap, which is a secure web application designed to support data capture real-time data entry validation and a de-identified data export mechanism to common statistical packages. The survey was sent via a web link to the patients. The survey was mailed to the patients who did not have access to the internet.

The dependent variable was the overall patient perception of the care they receive at the Heroes clinic (Question: CU Heroes Clinic dental students are able to relieve or cure most dental problems) for both the frameworks. This measure provides us with an overall perception of the patients about the care they receive from the dental students at the Heroes Clinic. This question was chosen as an outcome because the verbiage of the question helps us to understand if the patient has overall satisfaction from their treatment experience at the Heroes Clinic.

The independent variables for the SERVQUAL framework were the 5 subscales of empathy and 3 subscales of responsiveness. The independent variables for the DSQ framework were general satisfaction, 6 subscales of quality of care, 3 subscales of pain management, availability/convenience and 2 subscales of accessibility. The scaling of all the independent and outcome variables was changed from a 5-point Likert scale to binary. The initial Likert scale categorized the variables as 1 = strongly agree, 2 = agree, 3 = undecided, 4 = disagree and 5 = strongly disagree. Strongly agree and agree were then merged to form one category. Strongly disagree, disagree, and undecided were merged to form the second category. In addition, patient demographics were collected.

Demographic data were analyzed using a variety of descriptive statistics including percentages and means. In addition, scaled means of all the independent variables were computed. The scaled mean [16] was calculated by first calculating the overall mean score of the independent variable of interest (including all the items in the scale) and then dividing the resultant overall mean score by the number of items in that scale. The scaled mean score near 1.0 represents extreme dissatisfaction, scores near 3.0 represent neutrality and scores near 5.0 represent extreme satisfaction of the patients.

Fisher’s exact test was conducted to test any significant differences between the patients’ perception of the quality of care they receive at the Heroes clinic (outcome variable) and the SERVQUAL and DSQ independent variables. A significance level of *p* < 0.05 was used to test the differences. Bivariate analysis frequency, percentage, and *p*-value were reported. All the analyses were completed using SAS 9.4.

## Results

The survey was sent to 500 active patients at the clinic and 177 patients responded to the survey (35% response rate). Over 50% of responding patients were between the ages of 20–35 years and 78% were male (Table 2). Sixty-eight percent of the patients were Caucasian/White. Sixty-three percent of the veterans were students. Ninety-two percent of veterans reported their general health to be in good condition and 80% reported with good oral health. Fifty-two percent of the veterans were in the end phase and 48% were in the middle phase of treatment when they filled out the questionnaire.

**Table 2:** Demographics of survey respondents

Variables	Categories	Frequency (%) *N* = 177
Age	20–35	92 (51.98)
	36–50	27 (15.25)
	51–65	24 (13.56)
	66–80	30 (16.95)
	80+	4 (2.26)
Gender	Male	138 (78.41)
	Female	38 (21.59)
Student	Yes	112 (63.28)
	No	65 (36.72)
Military Branch	Air Force	32 (18.08)
	Army	60 (33.9)
	Coast Guard	3 (1.69)
	Marine	41 (23.16)
	Navy	41 (23.16)
Military Era	WWII	2 (1.15)
	Korea	2 (1.15)
	Vietnam	43 (24.71)
	Gulf/desert storm	19 (10.92)
	Post 9/11	108 (62.07)
Race	American Indian/Alaska Native	5 (2.85)
	Asian	2 (1.14)
	Black or African American	8 (4.57)
	Caucasian/White	119 (68.00)
	Native Hawaiian or Other Pacific Islander	0 (0)
	Hispanic/Latino	19 (10.86)
	Other	1 (0.57)
	Multiple	21 (12.00)
Health Oral Health	Excellent	43 (24.29)
	Very Good	79 (44.63)
	Good	43 (24.29)
	Fair	10 (5.65)
	Poor	2 (1.13)
	Excellent	17 (9.66)
Health Treatment Plan	Very Good	57 (32.39)
	Good	68 (38.64)
	Fair	24 (13.64)
	Poor	10 (5.68)

Table 3 presents the means, standard deviation, and scaled means for all the independent variables. The scaled means were closer to 5.0 for all the independent variables, which demonstrates high levels of empathy, responsiveness, general satisfaction, quality of care, and pain management provided by the dental care providers at Heroes clinic. In addition, the patients were highly satisfied with the accessibility and convenience of the services provided by the dental care providers at the Heroes clinic.

**Table 3:** Means for each variable (SERVQUAL and DSQ)

SERVQUAL	Mean (SD)	Scaled Mean
Empathy (5- items)	23.31*	4.66
Empathy 1: Explained Treatment	1.05 (0.22)	
Empathy 2: Administration Procedures	1.04 (0.20)	
Empathy 3: Listens to complaint	1.05 (0.22)	
Empathy 4: Registration Procedures	1.06 (0.23)	
Empathy 5: Knowledge about treatment needs	1.06 (0.23)	
Responsiveness (3 items)	14.03*	4.67
Responsiveness 1: Long time waiting	1.24 (0.43)	
Responsiveness 2: Issues related to care	1.08 (0.27)	
Responsiveness 3: Issues related to delays in care	1.17 (0.37)	
DSQ		
**General Satisfaction (1 item)	1.77 (0.43)	3.60
Quality of Care (6 items)	26.99*	4.49
Quality of Care 1: Careful examination	1.04 (0.20)	
Quality of Care 2: Respectful towards patients	1.02 (0.15)	
**Quality of Care 3: Thorough dental students	1.94 (0.24)	
Quality of Care 4: Explanatory about treatment procedures	1.03 (0.17)	
Quality of Care 5: Modern clinic	1.05 (0.21)	
**Quality of Care 6: Helpful dental students	1.91 (0.28)	
Pain Management (3 items)	11.41*	3.80
**Pain Management 1: Painless procedures	1.85 (0.36)	
**Pain Management 2: Efforts in reducing pain	1.88 (0.33)	
Pain Management 3: Patients not concerned about feeling pain	1.37 (0.49)	
Availability/Convenience (2 items)	7.73*	3.86
**Availability: Availability of more dentists	1.89 (0.32)	
Convenience: Conveniently located clinic	1.37 (0.48)	
Accessibility (2 items)	8.12*	4.06
**Accessibility 1: Appointment easily available	1.84 (0.36)	
Accessibility 2: Suitable office hours	1.22 (0.41)	
Outcome (1 item)	1.14 (0.35)	4.42

Number of respondents: 177

SD = Standard Deviation

**Items are coded in negative direction

*Potential range of mean scale: 6-items: 0–30; 5-items: 0–5, 3-items: 0–15, 2-items: 0–10

Table 4 describes the bivariate association results for the SERVQUAL data. It was found that the majority of the veterans were satisfied with the level of Empathy and Responsiveness provided by the dental student providers and the assistant director of the Heroes clinic. Eighty-three percent of the veterans agreed that the dental students gave personal attention in listening to their complaints (*p* = 0.022), were knowledgeable regarding their treatment needs (*p* = 0.006) and clearly explained all the treatment (*p* = 0.003) to them. Eighty-two percent agreed that the assistant director gave personal attention to registration procedures (*p* = 0.032) and responded promptly to issues and concerns related to their care (*p* < 0.0001). However, 75% of the veterans agreed that the assistant director responded promptly to issues and concerns related to delays in care (*p* = 0.0004). Additionally, 68% agreed that the assistant director responded promptly to long-time waiting (*p* = 0.011).

**Table 4:** Bivariate association of SERVQUAL independent variables with the primary outcome on a binary scale

Variables*	Frequency** (percentage) of respondents’ agreement	*P*-value**
Empathy 1: Explained Treatment	143 (83.43%)	0.003
Empathy 2: Administration Procedures	143 (82.66%)	0.203
Empathy 3: Listens to complaint	145 (82.86%)	0.022
Empathy 4: Registration Procedures	144 (82.29%)	0.032
Empathy 5: Knowledge about treatment needs	145 (82.86%)	0.006
Responsiveness 1: Long time waiting	117 (67.63%)	0.011
Responsiveness 2: Issues related to care	144 (82.29%)	< 0.0001
Responsiveness 3: Issues related to delays in care	130 (75.14%)	0.0004

Number of respondents: 177

*All the variables of interest were converted from a Likert scale (1 = strongly agree, 2 = agree, 3 = undecided, 4 = disagree, 5 = strongly disagree) to a binary scale (Strongly agree and agree combined to 1 = agree, and strongly disagree, disagree, undecided combined to 2 = disagree)

Primary Outcome: Heroes Clinic providers can relieve most problems

**Test: Fisher’s Exact test frequency and p-value

Table 5 describes the bivariate association results for the DSQ data. It was found that 69% of the veterans were satisfied with the dental care they receive at the CU Heroes clinic (*p* = 0.009). The majority of the veterans were satisfied with the quality of care provided by the dental student providers at the Heroes clinic. Eighty-one percent of the veterans agreed that dental students always treated them with respect (*p* = 0.0003). Eighty-four percent agreed that the dental students were very careful with their examination (*p* = 0.008) and clearly explained the procedure before beginning the treatment (*p* = 0.021). In addition to being satisfied by the Heroes clinic quality of care, 56% of the veterans agreed that they are not concerned about feeling pain when they visit Heroes clinic for receiving dental care (*p* = 0.022). Additionally, 78% agreed that dental students do everything to reduce their pain (*p* = 0.013). Furthermore, 83% of the veterans agreed that the Heroes clinic is very modern and up to date (*p* = 0.014).

**Table 5:** Bivariate association of DSQ independent variables with the primary outcome on a binary scale

Variables*	Frequency** (percentage) of respondents’ agreement	*P*-value**
***General Satisfaction	120 (68.57%)	0.009
Quality of Care 1: Careful examination	147 (84%)	0.008
Quality of Care 2: Respectful towards patients	150 (85.71%)	0.0003
***Quality of Care 3: Not so thorough dental students	8 (4.62%)	0.144
Quality of Care 4: Explanatory about treatment procedures	147 (84.48%)	0.021
Quality of Care 5: Modern clinic	147 (83.05%)	0.014
***Quality of Care 6: Not helpful dental students	11 (6.29%)	0.103
***Pain Management 1: Painful procedures	22 (12.57%)	0.228
***Pain Management 2: Efforts in reducing pain	135 (77.59%)	0.013
Pain Management 3: Patients not concerned about feeling pain	98 (56.32%)	0.022
***Availability: Lower availability of dentists	18 (10.34%)	0.247
Convenience: Conveniently located clinic	97 (55.43%)	0.123
***Accessibility 1: Appointment not easily available	26 (15.03%)	0.056
Accessibility 2: Suitable office hours	122 (69.71%)	0.015

Number of respondents: 177

***Items are coded in negative direction

*All the variables of interest were converted from a Likert scale (1 = strongly agree, 2 = agree, 3 = undecided, 4 = disagree, 5 = strongly disagree) to a binary scale (strongly agree and agree combined to 1 = agree, and strongly disagree, disagree, undecided combined to 2 = disagree)

Primary Outcome: Heroes Clinic providers can relieve most problems

**Test: Fisher’s Exact test frequency and p-value

## Discussion

This study demonstrates higher levels of empathy, responsiveness, and quality of care as reported by the veteran population visiting the CU’s Heroes Clinic. The results of this study found that the veterans visiting the Heroes clinic were satisfied with the quality of dental care received at the clinic.

The results of this study demonstrate that the overall patient perception of care in the Heroes clinic was dependent on the personal attention of the student provider. The patients agreed that the student providers were empathetic and carefully listened to their complaint. The patients also reported that the student providers were knowledgeable regarding their treatment needs, and clearly explained the treatment process before beginning the treatment in a respectful manner. Previous studies have revealed that the positive attitude of the dental care providers, including a detailed explanation of treatment, improves patient satisfaction level [17, 18]. The importance of these interpersonal factors (communication and explanation of treatment) for dental patient satisfaction was supported by two research studies conducted in Finland and the US [19, 20]. The study conducted in the US reported the dentist’s awareness of patient discomfort and explanation of treatment as the two most important issues. Similarly, another study conducted in the US reported personality and communication to be most important to satisfaction with dental care [21]. It has been seen that the patients prefer a caring and pleasant dentist as compared to a skilled one alone [22].

The orientation provided to the students in the form of *Heroes Clinic Expectations* document and the workshop held for faculty to train them in supervising the student providers at the Heroes clinic plays an important role in improving their skills to communicate with their patients and work collaboratively with other providers. The orientation communicates about the importance of a detailed explanation of treatment procedures and emphasis on needs of the patients in order to manage treatment-related anxiety and apprehensions, which some patients of the Heroes clinic may experience. The results of this study demonstrate higher satisfaction in patients of Heroes clinic in terms of quality of care, empathy, and responsiveness provided by student providers. The orientation assists student providers in better understanding the personal perspective of the patients thereby improving the quality of services provided by the student providers at the Heroes Clinic.

The authors decided to use both SERVQUAL and the DSQ surveys in this study because they wanted to capture several dimensions of patient-perceived overall dental care they receive at the Heroes clinic. SERVQUAL was originally used in the marketing industry to address gap analysis and the difference in expectations and perceptions of quality of service [11]. However, a handful of studies have observed the relevance of the SERVQUAL model to measure the service quality in the dental care setting [23, 24]. A few studies that have used SERVQUAL quality measures to measure the patient perception of overall care, reported the overall patient perception to be highly correlated with the dental care providers being empathetic and responsive [25, 26]. DSQ is a valid and reliable framework that has been used to measure dental satisfaction constructs by various studies [16, 27, 28]. A recent systematic review that reviewed the literature for over two decades reported that DSQ was used frequently to measure overall patient perception of care. However, none of the studies included in the review measured empathy of the provider, which is closely related to patients’ perception of care [29, 30]. The present study has used both the frameworks to create a better understanding of the overall perception of care received by the patient and its association with empathy, knowledge, responsiveness and active listening of the provider.

The study had some limitations. Although the response rate was on the lower side, 35%, it still provides reasonable patient feedback about the empathy and quality of care delivered at the Heroes Clinic. The authors speculate that a lower response rate was seen as most of the veterans receiving care at the Heroes clinic are students. All the patients were contacted via email or mail depending upon their preference to be contacted and reminders were sent three times. This method was used because we did not want to increase the time spent at the clinic by the patients. However, literature has shown that response to web or email surveys is lower than in-person surveys [31]. In addition, the inferences from this study should not be used as causal inferences because the study used a cross-sectional design.

## Conclusions

This evaluation demonstrated that the overall patient perception of care was contingent on the student provider’s empathy, communication style, knowledge about the treatments and respect for the patient. It is demonstrated that the patients provided with positive feedback and were satisfied with the quality of dental care provided at the Heroes clinic. Heroes Clinic has provided quality dental care to the veterans as attested by the patients.

## Data Availability

The datasets used and/or analyzed during the current study are available from the corresponding author on reasonable request.

## Abbreviations

CU: Colorado University
DSQ: Dental Satisfaction Questionnaire
SDM: School of Dental Medicine
SERVQUAL: Service Quality Measures
VA: Veterans’ Administration
